# STEREOTYPE EMBODIMENT AFFECTS FEAR OF FALLING: COMPARISON OF EXPLICIT AND IMPLICIT MEASURES

**DOI:** 10.1093/geroni/igae098.3725

**Published:** 2024-12-31

**Authors:** Amber Blount, Eman Ali, Carla Leinbach, Dahee Kim, Kworweinski Lafontant, Nichole Lighthall, Ladda Thiamwong

**Affiliations:** University of Central Florida, Orlando, Florida, United States; University of Central Florida, Orlando, Florida, United States; University of Central Florida, Orlando, Florida, United States; University of Central Florida, Orlando, Florida, United States; University of Central Florida, Orlando, Florida, United States; University of Central Florida, Orlando, Florida, United States; University of Central Florida, Orlando, Florida, United States

## Abstract

Stereotype Embodiment Theory posits that societal stereotypes about aging affect older adults’ self-perceptions of aging (SPA). The implicit belief of negative stereotype associations may be less influential than explicit stereotype beliefs. Stereotype affirming beliefs can be implicit in which there’s no self-identification, while stereotype embodiment is explicit self-identification with negative stereotypes. Little is known about the role of implicit associations of aging in older adults. Negative SPA is associated with fear of falling (FOF) in older adults. This study explored the association between stereotype embodiment and implicit associations, and FOF among low-income community-dwelling older adults (N=162, MeanAge = 74.6, SD = 7.15). Stereotype embodiment was assessed using the Consequences-control-negative subdomain of the Brief Ageing Perceptions Questionnaire, while implicit associations of aging was measured using the Age Implicit Association Test. FOF was assessed using the Short Fall Efficacy Scale-International. Most participants (86.9%) exhibited implicit aging stereotype beliefs, with 41.4% also displaying stereotype embodiment. Among those with both negative implicit associations and stereotype embodiment, 48.3% had high FOF. No significant correlation was found between stereotype embodiment and implicit associations. However, a significant association between stereotype embodiment and FOF was found (p < 0.05), whereas the association between implicit associations and FOF was non-significant. These findings support stereotype embodiment theory, highlighting that self-identification with negative aging stereotypes can contribute to self-fulfilling prophecies more than mere stereotype beliefs do. This holds implications for clinical gerontologists by indicating a need for interventions targeting maladaptive self-stereotyping to mitigate overestimated FOF in older adults.

